# Heterogeneous and Dynamic Prevalence of Asymptomatic Influenza Virus Infections

**DOI:** 10.3201/eid2405.180075

**Published:** 2018-05

**Authors:** Luis Furuya-Kanamori, Laith Yakob

**Affiliations:** Qatar University, Doha, Qatar (L. Furuya-Kanamori);; Australian National University, Canberra, Australian Capital Territory, Australia (L. Furuya-Kanamori);; London School of Hygiene & Tropical Medicine, London, UK (L. Yakob)

**Keywords:** epidemiology, influenza virus, viruses, asymptomatic infections, subclinical infections, influenza

**In Response:** We thank Leung and Cowling (*1*) for taking time to comment on our article (*2*). One problem with the random effects model is the rapid decline in performance of the model as the heterogeneity within studies increases. Extensive heterogeneity for asymptomatic (Ι^2^ = 97%; Τ^2^ = 0.31) and subclinical (Ι^2^ = 97%; Τ^2^ = 0.45) infection was identified. However, the model selected to pool the prevalence estimates—inverse variance heterogeneity—maintains its coverage at the nominal level, even when large heterogeneity is present (*3*).

Regarding inclusion criteria, we elected to review all publications detailing asymptomatic influenza prevalence in humans, as is made clear from the original article’s title onward. This method included experimental studies, as well as newly emerging zoonotic strains. We note further that the 2 experimental studies in our review had subclinical influenza infection levels within the range identified in the pooled estimate of the meta-analysis (43.4%, 95% CI 25.4%–61.8%). Also, because antibody titers can vary drastically with technique used and between laboratories, we used the antibody titer threshold defined by each individual study.

The results/conclusions from the study published by Leung et al. (*4*) cannot be compared with those reported in our meta-analysis (*2*) for 2 important reasons. First, the case definition for asymptomatic was different; Leung et al. grouped patients without signs and symptoms (asymptomatic in our meta-analysis) with patients that did not fulfill the criteria of influenza-like illness (subclinical in our meta-analysis). We explained in our article why pooling asymptomatic and subclinical cases is inappropriate and likely to provide spurious results. As an example of how the case definition can affect the results, Pascalis et al. found that in the same group of patients, 30.6% had subclinical infection (not fulfilling criteria for influenza-like illness) but only 1.6% had no symptoms at all (*5*). Second, the number of studies included in the 2 meta-analyses was different: our comprehensive review comprised 55 studies, whereas Leung et al. included a subset of only 30 studies pertaining specifically to seasonal influenza. The different studies included and different meta-analytical methods unsurprisingly yielded different outcomes.
